# Systems Biology Analysis of Temporal *In vivo Brucella melitensis* and Bovine Transcriptomes Predicts host:Pathogen Protein–Protein Interactions

**DOI:** 10.3389/fmicb.2017.01275

**Published:** 2017-07-27

**Authors:** Carlos A. Rossetti, Kenneth L. Drake, Sara D. Lawhon, Jairo S. Nunes, Tamara Gull, Sangeeta Khare, Leslie G. Adams

**Affiliations:** ^1^Department of Veterinary Pathobiology, College of Veterinary Medicine and Biomedical Science, Texas A&M University College Station, TX, United States; ^2^Seralogix, Inc. Austin, TX, United States

**Keywords:** interactome model, Bayesian analysis, virulence factors, Peyer's patch

## Abstract

To date, fewer than 200 gene-products have been identified as *Brucella* virulence factors, and most were characterized individually without considering how they are temporally and coordinately expressed or secreted during the infection process. Here, we describe and analyze the *in vivo* temporal transcriptional profile of *Brucella melitensis* during the initial 4 h interaction with cattle. Pathway analysis revealed an activation of the “Two component system” providing evidence that the *in vivo Brucella* sense and actively regulate their metabolism through the transition to an intracellular lifestyle. Contrarily, other *Brucella* pathways involved in virulence such as “ABC transporters” and “T4SS system” were repressed suggesting a silencing strategy to avoid stimulation of the host innate immune response very early in the infection process. Also, three flagellum-encoded loci (BMEII0150-0168, BMEII1080-1089, and BMEII1105-1114), the “flagellar assembly” pathway and the cell components “bacterial-type flagellum hook” and “bacterial-type flagellum” were repressed in the tissue-associated *B. melitensis*, while *RopE1* sigma factor, a flagellar repressor, was activated throughout the experiment. These results support the idea that *Brucella* employ a stealthy strategy at the onset of the infection of susceptible hosts. Further, through systems-level *in silico* host:pathogen protein–protein interactions simulation and correlation of pathogen gene expression with the host gene perturbations, we identified unanticipated interactions such as VirB11::MAPK8IP1; BtaE::NFKBIA, and 22 kDa OMP precursor::BAD and MAP2K3. These findings are suggestive of new virulence factors and mechanisms responsible for *Brucella* evasion of the host's protective immune response and the capability to maintain a dormant state. The predicted protein–protein interactions and the points of disruption provide novel insights that will stimulate advanced hypothesis-driven approaches toward revealing a clearer understanding of new virulence factors and mechanisms influencing the pathogenesis of brucellosis.

## Introduction

*Brucella*, an aerobic non-motile Gram-negative coccobacillus, is the etiological agent of brucellosis, a worldwide anthropozoonotic infectious disease that causes chronic infections with persistent or recurrent bacteremia in susceptible hosts, and mid- to late gestation abortion in pregnant animals. At present, there are 11 recognized species within the genus *Brucella* based on preferential host specificity (O'Callaghan and Whatmore, 2011; Whatmore et al., 2014). Goats and sheep are the preferred hosts for *Brucella melitensis* (Alton, 1990), although this pathogen also infects cattle depending on specific epidemiological conditions (Kalher, 2000; Banai, 2010; Alvarez et al., 2011; Liu et al., 2012; Wareth et al., 2014). *B. melitensis* is also the most pathogenic and the most frequent causative agent of brucellosis in humans (Traxler et al., 2013). Clinically, human brucellosis can be an incapacitating disease that results in intermittent fever, chills, sweats, weakness, myalgia, osteoarticular complications, endocarditis, depression, and anorexia with low mortality (Dean et al., 2012).

The predominant route for *B. melitensis* penetration after natural exposure is the alimentary tract (Adams, 2002). Usually *B. melitensis* enters through the oral mucosa and colonizes the lymph nodes that drain the facial area (Carpenter, 1924; von Bargen et al., 2015); although several studies have isolated *Brucella* from different sections of the alimentary tract and feces revealing the possibility that brucellae invade in multiple sites of the gastrointestinal tract (Carpenter, 1924; Davis et al., 1988). We previously found that under experimental conditions, *B. melitensis* was able to invade the bovine host through the domed epithelium of jejuno-ileal Peyer's patches followed by rapid systemic dissemination (Rossetti et al., 2013). The calf ligated jejuno-ileal loop model has been demonstrated to be a very useful model to study *in vivo* natural host:infectious agent molecular and morphological initial interactions (Santos et al., 2002; Khare et al., 2009, 2012; Winter et al., 2010; Lawhon et al., 2011; Rossetti et al., 2013), a subject that has not been broadly studied in brucellosis.

*Brucella* lack several classical bacterial virulence factors such as exotoxins, cytolysins, a capsule, fimbriae, plasmids, resistant strains, lysogenic phages, antigenic variation, or endotoxic lipopolysaccharide among others (Moreno and Moriyón, 2002). *Brucella* has developed a stealthy strategy to avoid being recognized and successfully infect hosts. Succinctly stated, *Brucella* circumvents strong innate immune responses, obstructs the direct action of bactericidal substances, resists destruction by professional phagocytes and maintains the host cells alive to establish long lasting infections (Barquero-Calvo et al., 2007). Although, significant advances have been made lately (de Figueiredo et al., 2015), the molecular pathogenesis of brucellosis is still incompletely understood. To date, fewer than 200 gene products have been identified as *Brucella* virulence factors (He, 2012), and very few related to adhesion and invasion function have been characterized. For instance, the heat shock protein 60 (Hsp60) family proteins on the surface of *Brucella abortus* have been found to bind macrophage and intestinal M cells cellular prion protein (PrP^c^) before internalization (Watarai et al., 2003; Nakato et al., 2012). The SP41 (UgpB) protein that has significant homology with the glycerol-3-phosphate-binding ABC transporter protein interacts with cellular sialic acid residues, facilitating efficient host invasion (Castaneda-Roldán et al., 2006). Other recently characterized proteins in the brucellae genome, such as, BmaC (Posadas et al., 2012), BtaE (Ruiz-Ranwez et al., 2013a), BtaF (Ruiz-Ranwez et al., 2013b) interact with different components of the extracellular matrix, while novel adhesion-encoding regions *inv* (Alva-Perez et al., 2014), or BigA (Czibener et al., 2016) have been demonstrated to promote adhesion and invasion, but their target host molecules have not been identified yet.

A well-recognized *Brucella* virulence factor is the two-component response regulator, BvrR/BvrS, that modulates the host cell cytoskeleton upon *Brucella* invasion (Sola-Landa et al., 1998) and regulates the *Brucella* OMP expression (Guzmán-Verri et al., 2002). Dysfunction of BvrR/S response regulator system induces mutant strains with reduced invasiveness and failure to replicate and survive intracellularly. *Brucella* lipopolysaccharide is also a confirmed virulence factor (Lapaque et al., 2005), that prevents complement-mediated bacterial killing (Allen et al., 1998; Tumurkhuu et al., 2006), provides resistance against antimicrobial peptides such as defensins, lysozyme, and lactorerrin (Martínez de Tejada et al., 1995) and inhibits cell death (Pei and Ficht, 2004; Pei et al., 2006). Additionally, *Brucella* LPS masks recognition of the pathogen-associated molecular patterns (PAMPs) from immune-receptor recognition, and as a consequence, impedes, or attenuates proinflammatory responses and immune system activation (Forestier et al., 2000; Jiménez de Bagués et al., 2004). Simultaneously, the type four secretion system (T4SS) is also a key virulence factor for *Brucella* intracellular survival (O'Callaghan et al., 1999), persistent infection in mice and induction of the host immune response (Rolan and Tsolis, 2007; Roux et al., 2007). This virulence factor, encoded by a *virB* operon, is induced by early phagosome acidification after phagocytosis (Boschiroli et al., 2002; Celli et al., 2003) to translocate effector molecules directly into the host cell cytoplasm. Additional investigations have identified several of these effector proteins, although most of their functions remain undefined (de Jong et al., 2008, 2013; de Barsy et al., 2011; Marchesini et al., 2011, 2016; Salcedo et al., 2013; Ke et al., 2015; Del Giudice et al., 2016). The secretion systems and secretomes of *Brucella* were recently computationally analyzed, resulting in the prediction of 29 host-pathogen specific interactions between cattle and *B. abortus* and 36 host-pathogen interactions between sheep and *B. melitensis* proteins (Sankarasubramanian et al., 2016). The two-component system RegB/A, including the *aceA* encoding isocitrate lysase, has been found to play a critical role in the persistence and *in vivo* pathogenicity of *Brucella suis* (Abdou et al., 2017).

An additional virulence mechanism used by *Brucella* to survive intracellularly is the periplasmic compound cyclic B-1,2 glucan, that interferes with cellular trafficking and maturation of the *Brucella*-containing vacuole by disrupting cholesterol-rich lipid rafts present on phagosomal membranes and preventing the phagosome-lysosome fusion (Arellano-Reynoso et al., 2005; Martirosyan et al., 2012). Other virulence elements reported to sustain a chronic infection include: phosphatidylcholine, a phospholipid compound abundant in eukaryotic cell membranes that facilitate *Brucella* avoidance of host recognition (Comerci et al., 2006; Conde-Alvarez et al., 2006); PrpA, a proline-racemase family compound that elicits B lymphocyte polyclonal activation (Spera et al., 2006); BtpA and BtpB, *Brucella* TIR-containing effector proteins that suppress innate immunity and modulate host inflammatory responses during infection (Salcedo et al., 2008, 2013); MucR, a transcriptional regulator involved in *Brucella* metabolism, cell wall/envelope biogenesis, replication, type IV secretion system, quorum sensing system, and stress tolerance (Dong et al., 2013) and a flagellar appendage, required for virulence in a mouse infection model (Fretin et al., 2005).

Most of these virulence factors were characterized individually without considering how they are temporally and coordinately expressed or secreted during the infection process. Recently, several experiments have been performed to more fully understand the sequential expression and coordinated regulation of the infection process (Kohler et al., 2002; Al Dahouk et al., 2008; Rambow-Larsen et al., 2008; Lamontagne et al., 2009; Rossetti et al., 2009; Wang et al., 2009; Viadas et al., 2010; Weeks et al., 2010; Hanna et al., 2013; Tian et al., 2013). These experiments using *in vitro* culture media, infected cell cultures, or infected mice were successful for generating initial hypotheses to enhance the understanding of the pathogenesis of brucellosis.

Here, we describe an integrative approach of experimentation and computation to analyze the *in vivo* temporal transcriptional profile of *B. melitensis* during the first 4 h of the interaction with a naturally susceptible host, using the established calf jejuno-ileal loop model of infection. We then performed a system-level analysis by applying both a traditional statistical differential analysis to determine significance of *B. melitensis* gene expression and a pathway and gene ontology (GO) analysis that employed a dynamic Bayesian network (DBN) technique (Khare et al., 2016) to identify perturbations trends over time. The fundamental concept of systems biology is to: (1) perturb a system—(time-course *B. melitensis* infected bovine Peyer's patch), (2) measure systems-wide responses—(*B. melitensis* and bovine transcriptomes), and (3) integrate measured responses into a model—(host:pathogen::Bovine:*B*. melitensis interactome model) to understand the observations and iteratively predict novel interactions and perturbations. The system-level analyses aided understanding of the strategies exploited by *B. melitensis* to invade, survive, and replicate intracellularly; and to identify perturbations of major genes modulating critical cellular pathways in the pathogenesis of brucellosis. Further, through systems-level *in silico* host-pathogen protein–protein interactions (PPIs) simulation (see File S1), we were able to make inferred predictions of interactions of close apposition with specific *B. melitensis* expressed genes/proteins to plausible host (bovine) pathway points of disruption or perturbations. The predicted PPIs and the points of disruption provide novel insights that will stimulate advanced iterative hypothesis-driven approaches toward revealing a clearer understanding of new virulence factors and mechanisms contributing to the evasion of the host's protective immune responses.

## Materials and methods

### Infection model

The *in vivo* infection model for *Brucella* was described previously (Rossetti et al., 2013). Briefly, five bovine jejuno-ileal segments from four calves were inoculated intraluminally with 3 ml of a suspension containing 1 × 10^9^ CFU of *B. melitensis* 16 M/ml (total of 3 × 10^9^ CFU) at late-log growth phase cultured in F12K medium (ATCC®) supplemented with 10% heat-inactivated fetal bovine serum (HI-FBS) (ATCC®). One infected segment was removed at every time point (0.25, 0.5, 1, 2, 4 h post-inoculation from each of the four calves.), and six to ten 6 mm biopsy punches were collected from each segment. The mucosal layer of Peyer's patch was immediately dissected, macerated and homogenized in TRI-Reagent® (Ambion, Austin, TX). Subsequently, samples were appropriately contained and transported to an inspected and approved BSL-3 laboratory for immediate RNA extraction. Calves were euthanized with an intravenous bolus of sodium pentobarbital at the completion of the procedures. All animal experiments were approved by the Texas A&M University Institutional Animal Care and Research Advisory Committee (AUP#2003-178). Surgeries were performed under biosecurity laboratory III (BSL-3) conditions in CDC-approved isolation buildings at the Texas A&M University experimental farm (College Station, TX).

### RNA isolation, labeling, and hybridization

RNA isolation, labeling, and hybridization procedures were performed as described in previous experiments (Rossetti et al., 2010, 2011b). Total RNA from *B. melitensis*-infected bovine Peyer's patches was extracted according to the TRI-Reagent manufacturer's instructions. Tissue-associated *B. melitensis* total RNA was initially enriched (MICROBEnrich®, Ambion) and then amplified from 30 μg of total RNA from *B. melitensis*-infected bovine Peyer's patches (Rossetti et al., 2010). Briefly, the enriched RNA was precipitated in 100% ethanol at −20°C, washed and re-suspended in 25 μl of DEPC-treated water (Ambion). Immediately, the total amount of RNA was linearly amplified in a 3 step-protocol. First, RNA was reverse transcribed to cDNA using *B. melitensis* genome direct primers (BmGDPs), T7 promoter-template switching primer (T7-TS) (Sigma Genosys, The Woodland, TX) and Moloney Murine Leukemia Virus Reverse Transcriptase (Clontech, Palo Alto, CA). In the next step, the second-strand cDNA was synthesized and purified (Qiagen, Valencia, CA), followed by concentration in a speed-vac with no heat. In the last step, the *in vitro* transcription, was performed using the double-stranded cDNA as the template and T7 polymerase (Ambion). Then, 10 μg of each experimental sample (*n* = 44, i.e., 4 were *in vitro*-grown cultures of *B. melitensis* at late-log phase of growth; 20 were enriched and amplified *B. melitensis* RNA from total RNA from infected bovine Peyer's patches; and an additional 20 were from total RNA of infected bovine Peyer's patches) were reverse transcribed overnight to amino-allyl cDNA using 1.5 ug of *B. melitensis* genomic directed primers (*Bm*GDPs) (Rossetti et al., 2010), labeled with Cy3 (Amersham Pharmacia Biosciences, Piscataway, NJ), mixed with 0.5 μg of Cy5 labeled *B. melitensis* gDNA, and applied to a custom 3.2K *B. melitensis* oligoarray (Weeks et al., 2010). Since the enrichment procedure does not eliminate host RNA, total RNA from *B. melitensis*-infected bovine Peyer's patches were also reverse transcribed, labeled and hybridized on *B. melitensis* oligo microarray, due to a potential concern that eukaryotic RNA present in enriched and amplified samples could possibly overlap with sequences of the *B. melitensis* transcripts and cross hybridized with probes on *B. melitensis* oligo microarrays, resulting in falsely detected pathogen genes. The isolation and labeling of *B. melitensis* gDNA has been described in detail elsewhere (Rossetti et al., 2009). Slides were hybridized at 45°C for approximately 20 h in a dark humid chamber (Corning, Corning, NY). Then, washed for 10 min at hybridization temperature with low stringency buffer [1X SSC, 0.2% SDS] followed by two 5-min washes with a higher stringency buffer [0.1X SSC, 0.2% SDS and 0.1X SSC] at room temperature with mild agitation, dried by centrifugation and immediately scanned.

### Data acquisition, normalization, and microarray data analysis

Immediately after washing, the dried slides were scanned using a commercial laser scanner (GenePix 4100; Axon Instruments Inc., Foster City, CA). Scans were performed using the autoscan feature with the percentage of saturated pixels set at 0.03%. The genes represented on the arrays were adjusted for background and normalized to internal controls using image analysis software (GenePixPro 6.0; Axon Instruments Inc.). Genes with fluorescent signal values below background were disregarded in all analyses. Arrays were initially normalized against *B. melitensis* genomic DNA, and the resulting data were analyzed and modeled using an integrated platform termed the BioSignature Discovery System (BioSignatureDS®) (Seralogix, LLC, Austin, TX; http://www.seralogix.com) explained in detail elsewhere (Lawhon et al., 2011; Rossetti et al., 2013). The tissue-associated *B. melitensis* gene expression at every time point (0.25–4 h) was compared to the gene expression of the inoculum (i.e., *in vitro*-grown cultures of *B. melitensis* at late-log phase of growth, cultured in F12K medium with 10% HI-FBS; *n* = 4). Significantly expressed genes were determined with the *z*-test (*p* < 0.025) (enhanced with Bayesian methods of variance estimation) after subtracting those genes also expressed at statistically significant levels when total RNA of *B. melitensis*-infected bovine Peyer's patches was compared to the gene expression of the inoculum. BiosignatureDS tools for statistical *z*-score gene thresholding, *Brucella* pathway and gene ontology (GO) perturbation scoring (scored using Bayesian Information Criterion and transformed to *z*-score), and mechanistic gene identification were used for the comprehensive analysis performed in this study. A specialized application was developed to implement algorithms that integrate multiple sources of prior biological knowledge (PBK) into the inference of host-pathogen protein–protein interaction (PPIs) prediction (see File S1 for complete details). Briefly, we adopted three algorithmic methods for the identification of candidate interaction points for use in network learning between the host and the pathogen from *in vivo* gene expression data. These algorithmic methods were: (1) a sequence-similarity interaction transference procedure; (2) structural protein domain-based algorithm; and (3) a functional gene-ontology-based algorithm. Gene candidates for inclusion in our interaction prediction process were selected based on interpretation of pathway and GO analyses conducted by our Dynamic Bayesian Network methodology. The *B. melitensis* gene transcriptome was employed and ≈600 bovine host genes selected from 12 perturbed and immune response relevant pathways and 10 GO terms to form gene sets representing two “unconnected” system models for starting the “interactome model” network learning process. Those algorithms yielded 348, 68, and 295 potential host-pathogen PPIs, respectively, that comprised the set of potential interactions at the interface of the pathogen and host systems. These potential interactions were then included into the Bayesian host-pathogen network structure learning algorithm. The method employs model structures to initialize learning with biologically relevant structures and utilize actual time-course co-expressed gene and other “omic” data from pathogen and host to search for a set of structures in which the data best fit. Microarray data and metadata are deposited in the Gene Expression Omnibus at the National Center for Biotechnology Information (http://www.ncbi.nlm.nih.gov/geo/) Accession #GSE89053.

For microarray results validation, six randomly selected genes with consistently differential expressed from 15 min to 4 h post-infection (p.i.) by microarray results, were analyzed at every time point by quantitative RT-PCR (qRT-PCR) following the protocol described elsewhere (Rossetti et al., 2011b). Primers (Sigma Genosys) of tested genes were designed by Primer Express Software v2.0 (Applied Biosystems) (Table S1). For each gene tested, the individual calculated threshold cycles (Ct) were averaged among each condition and normalized to the Ct of the 16S rRNA gene from the same cDNA samples before calculating the fold change using the ΔΔCt method (Livak and Schmittgen, 2001). For each primer pair, a negative control (water) and an RNA sample without reverse transcriptase (to determine genomic DNA contamination) were included as controls during cDNA quantitation. Statistical significance was determined by Student's *t*-test and expression differences considered significant when *P* < 0.05. As gene expression by microarray and qRT-PCR were based on *z*-score and fold-change, respectively, array data were considered valid if the fold change of each gene tested by qRT-PCR was expressed in the same direction as determined by microarray analysis.

## Results

### *B. melitensis* transcriptome is perturbed at the onset of the infection process

**Figure 1:**
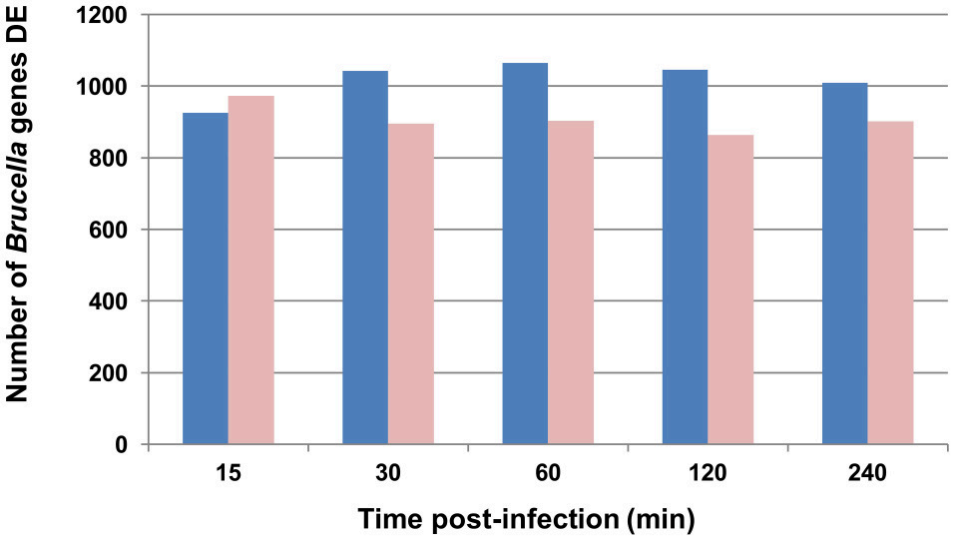
Graphic representation of *B. melitensis* genes differentially expressed (DE) throughout the experiment. Blue bars represent genes activated; light red bars represent genes repressed. For differential analysis, the *in vivo* infected loop gene expression is compared to the *in vitro* log growth phase inoculum as the control.

We previously reported that the number of *B. melitensis* 16M organisms after intraluminal inoculation increased from 15 min to 4 h p.i. (Rossetti et al., 2013) in this model. To study pathogen alterations in gene expression, pathway, and GO perturbations during the initial infection process, *Brucella* RNAs extracted from infected bovine Peyer's patches at different times p.i. were hybridized on *B. melitensis* microarrays and analyzed. As expected, the traditional *z*-score analysis (|2.24|, 97.5% confidence) identified a total of 2,356 different *B. melitensis* genes (1,221 up-regulated vs. 1,135 down-regulated) differentially expressed (DE) at least once over the 4 h time course p.i., compared to the *in vitro* grown control (Table S2). As opposed to the 1 h-peak of the host gene expression after infection (Rossetti et al., 2013), the total number of perturbed *Brucella* genes is rather constant in the first 4 h p.i. (15 min: 1,899 genes, 30 min: 1,937 genes, 1 h: 1,968 genes, 2 h: 1,909 genes and 4 h: 1,912 genes; Figure 1). The combined analysis of these results, i.e., the host and pathogen transcriptional profiles during bovine Peyer patch infection, clearly indicate that both host and *Brucella* gene expression responses are markedly perturbed at a very early time post-interaction. This is in concordance with other results (He et al., 2006; Rossetti et al., 2011b, 2012), which corroborates an initial transcriptionally perturbed period followed by a more quiescent one.

A group of 1,740 genes (55% of *B. melitensis* genome) was markedly perturbed in the same direction in at least 4 of 5 time points (Table S3). These genes were considered as the core set of genes associated with the adaptive changes of *B. melitensis* during the early *in vivo* bovine Peyer's patch infection, and therefore important in understanding key events in the early modulation of host response. From this set of 1,740 Differentially Expressed (DE) genes, 925 (53%) were activated and 815 (47%) were repressed compared with the *in vitro* grown culture. Interestingly, genes from the core set located on chromosome I were mainly activated (over 1,174 DE genes: 752 were up- and 422 were down-regulated), while genes located on chromosome II were mainly repressed in higher numbers (566 total DE genes: 173 were up- and 393 were down-regulated). Chromosome I encodes the majority of the core metabolic machinery for transcription, translation and protein synthesis, and Chromosome II is overrepresented in genes involved in pathways for utilization of specific substrates (membrane transport, central intermediary and energy metabolism, and regulation; Paulsen et al., 2002). Altogether, these results suggest that *Brucella* may restrain metabolic functions while inducing transcriptomic modifications to adapt from an extracellular to an intracellular lifestyle. These results are largely in concordance with previous publications (Lamontagne et al., 2009; Rossetti et al., 2011a,b) even though our study analyzes the complexity of *in vivo* invasion process in comparison with pathogen gene expression in other *in vitro* (i.e., one cell line) models of invasion.

Microarray gene expression data were validated by qRT-PCR. Six randomly selected *Brucella* genes, determined to be significantly affected throughout the first 4 h p.i., were chosen for verification at every time point (i.e., 30 data points). As shown by the representative examples in Figure S1, gene expression changes were consistent between microarray and qRT-PCR for genes with increased expression or genes with decreased expression relative to the control.

## Discussion

### Major *Brucella* virulence factors are down regulated at the onset of the infection

Within 15–30 min of *in vivo* exposure, *B. melitensis* adhere and immediately penetrate through the intestinal mucosa and Peyer's patch and rapidly disseminate through systemic circulation (Rossetti et al., 2013). Early stage host gene expression of Syndecan 2, Integrin alpha L and Integrin beta 2 genes coincide with initial *Brucella* adhesion which is coupled with simultaneous repression of two intestinal barrier-related pathways (Tight Junction and Trefoil Factors Initiated Mucosal Healing), subverting mucosal epithelial barrier function and facilitating *Brucella* transepithelial migration (Rossetti et al., 2013). To elucidate *Brucella* virulence mechanisms responsible for this host molecular response, we expanded our analysis on pathogen pathways (Table 1) and GO alterations (Tables S4–S8). Note that pathway molecular interactions and annotations are based on those provided by the Kyoto Encyclopedia of Genes and Genomes (KEGG; Kanehisa et al., 2017). Pathways and Gene Ontology groups are comprised of gene sets which may be either activated or repressed in some combination over time. The Bayesian scoring method computes the log-likelihood of the *in vivo* expressed data and measures its goodness-of-fit to a model trained with control data (the *in vitro* inoculum expression data). In this manner, it is possible to determine if a pathway or GO group is activated or repressed. In our computational system biology approach, if the sum of the individual gene scores within a pathway/GO group is positive then the pathway/GO score is considered to be activated. Otherwise, if the sum is negative, the pathway/GO score is considered repressed and assigned a negative score value. Table 1 shows the results of the pathway analysis scoring listed by pathway categories and sorted by activated or repressed state on the 15 min p.i. column. Specific gene expression scores within these pathways are provided in Table S7. Early in the infection process, the pathway category “Environmental Information Processing” has several important pathways involved in *B. melitensis* pathogenicity which are repressed at 15 min p.i. that include “Type IV secretion system,” “Type III secretion system,” Two-component system,” and ABC transporters. It is interesting to note that the Two-component regulatory systems (TCRSs) and Type III secretion system pathways reverse to an activated state at 30 min. p.i. In the cellular processes category, the “Flagellar assembly” and “Bacterial chemotaxis” pathways are also repressed across all time point p.i.

**Table 1:** Significantly perturbed pathways (Bayesian z-score >|2.24|) of tissue-associated *B. melitensis* during the first 4 h post-bovine Peyer's patch infection.

**KEGG Name**	**Description**	**T15**	**T30**	**T60**	**T120**	**T240**
**ENVIRONMENTAL INFORMATION PROCESSING**						
bme03010	Ribosome	9.63	8.38	11.27	9.53	9.72
bme03020	RNA polymerase	7.64	7.74	10.42	7.9	7.77
bme02060	Phosphotransferase system (PTS)	6.98	6.04	7.9	7.28	6.06
bme00970	Aminoacyl-tRNA biosynthesis	6.82	7.87	9.9	5.6	9.67
bme03060	Protein export	5.29	7.02	8.57	4.61	6.95
bme03410	Base excision repair	−7.33	−5.36	−5.4	−6.28	−5.82
bme03070 bme_M00333^*^	Type IV secretion system module	−7.67	−9.04	−8.87	−9.52	−6.86
bme03030	DNA replication	−9.41	−6.39	−6.62	−6.69	−6.22
bme02020	Two-component regulatory system	−9.55	8.66	8.65	6.74	9.52
bme02010	ABC transporters	−10.01	−8.34	−9.17	−6.44	−9.83
**GLYCAN BIOSYNTHESIS AND METABOLISM**						
bme00510 (map00510)^*^	N-Glycan biosynthesis	−8.34	5.1	7.9	6.02	6.43
bme00512 (map00512)^*^	O-Glycan biosynthesis	−9.82	−4.26	−4.96	−4.6	−5.59
bme00603	Glycosphingolipid biosynthesis	−9.99	4.81	−6.82	−5.03	−5.18
bme00604 (map00604)^*^	Glycosphingolipid biosynthesis	−9	−4.92	−6.56	−4.98	−5.46
bme00940 (map00940)^*^	Phenylpropanoid biosynthesis	−4.74	5.51	6.87	7.05	5.98
**CELLULAR PROCESSES**						
bme02030	Bacterial chemotaxis	−8.75	−8.68	−7.73	−7.93	−8.25
bme02040	Flagellar assembly	−9.21	−8.29	−9.01	−8.63	−7.17
**CARBOHYDRATE METABOLISM**						
bme00010	Glycolysis/Gluconeogenesis	8.34	7.05	7.27	6.89	8.52
**LIPID METABOLISM**						
bme00061	Fatty acid biosynthesis	7.3	7.63	8.34	6.07	7
**METABOLISM**						
bme00400	Phenylalanine, tyrosine and tryptophan biosynthesis	11.71	9.3	10.01	10.44	9.12
bme00230	Purine metabolism	10.38	9.05	11.96	7.56	8.2
bme00300	Lysine biosynthesis	8.98	6.83	6.77	6.54	5.84
bme00710 (map00710)^*^	Carbon fixation in photosynthetic organisms	8.89	6.6	6.92	6.42	7.81
bme00620	Pyruvate metabolism	8.66	7.41	8.59	5.99	6.42
bme00950 (map00950)^*^	Alkaloid biosynthesis I	8.35	7.48	7.04	7.44	6.31
bme00240	Pyrimidine metabolism	8.24	8.72	9.54	7.76	7.26
bme00190	Oxidative phosphorylation	8.09	7.53	10.17	7.87	7.72
bme00271 (map00270)^**^	Methionine metabolism	8	11.81	9.18	6.73	7.93
bme00740	Riboflavin metabolism	7.49	5.78	10.7	4.36	7.27
bme00720 (map00720)^*^	Reductive carboxylate cycle (CO2 fixation)	7.44	7.79	8.29	6.06	8.31
bme00020	Citrate cycle (TCA cycle)	7.43	8.42	8.95	7.39	8.41
bme00030	Pentose phosphate pathway	7.43	6.62	9.06	6.32	8.85
bme00790	Folate biosynthesis	7.43	6.17	10.52	4.84	7.62
bme00670	One carbon pool by folate	7.29	8.14	10.94	6.59	7.09
bme00251 (map00250)^**^	Glutamate metabolism	7.19	7.46	8.66	7.82	8.2
bme00760	Nicotinate and nicotinamide metabolism	7.05	6.86	6.85	6.17	8.13
bme00904 (map00904)^*^	Diterpenoid biosynthesis	6.95	4.35	6.81	3.82	5.08
bme00100 (map00100)^*^	Biosynthesis of steroids	6.91	6.47	6.88	4.4	6.29
bme00660	C5-Branched dibasic acid metabolism	6.89	7.59	8.19	6.1	6.94
bme00290	Valine, leucine and isoleucine biosynthesis	6.87	7.76	10.18	5.58	5.41
bme00770	Pantothenate and CoA biosynthesis	6.71	10.04	8.42	7.16	7.99
bme00680	Methane metabolism	6.69	7.93	10.68	9.28	7.01
bme00330	Arginine and proline metabolism	6.68	6.67	8.62	5.55	8.6
bme00621 (map00621)^*^	Biphenyl degradation	6.62	7.19	8.83	4.13	6.7
bme00730	Thiamine metabolism	6.5	8.39	7.51	5.52	6.54
bme00252 (map00250)^**^	Alanine and aspartate metabolism	6.36	6.19	8.28	4.05	9.89
bme00072	Synthesis and degradation of ketone bodies	6.26	6.72	9.33	5.34	9
bme00540	Lipopolysaccharide biosynthesis	6.11	5.42	5.98	3	4.94
bme00622	Toluene and xylene degradation	5.95	5.48	5.55	5.36	5
bme00460	Cyanoamino acid metabolism	5.67	6.33	6.96	3.49	5.85
bme00361	Gamma-Hexachlorocyclohexane degradation	5.32	7.54	7.83	5.58	7.45
bme00627	1,4-Dichlorobenzene degradation	5.32	5.1	5.23	5.01	4.73
bme00272 (map00270)^**^	Cysteine metabolism	5.29	8.32	7.05	5.43	9.11
bme00530 (map00530)^*^	Aminosugars metabolism	5.15	6	6.99	5.76	7.79
bme00983 (map00983)^*^	Drug metabolism - other enzymes	4.48	6.72	7.03	7.96	5.98
bme00900	Terpenoid biosynthesis	4.47	5.65	4.86	5.82	7.32
bme00643	Styrene degradation	−2.57	3.65	6.06	3.29	3.13
bme00062 (map00062)	Fatty acid elongation in mitochondria	−3.85	−5.45	−5.31	−4.04	−4.5
bme00472	D-Arginine and D-ornithine metabolism	−4.22	2.97	−3.66	−2.55	−3.76
bme00473	D-Alanine metabolism	−4.56	3.58	−6.23	−5.01	−5.55
bme00785	Lipoic acid metabolism	−4.73	−6.11	−4.56	−3.39	−3.99
bme01053	Biosynthesis of siderophore group nonribosomal peptides	−5.02	−7.26	−6.39	−5.46	8.37
bme00521	Streptomycin biosynthesis	−5.57	−8.27	−6.95	−6.95	−6.03
bme00523	Polyketide sugar unit biosynthesis	−5.68	−4.98	−7.14	−5.33	−6.09
bme00562 (map00562)^*^	Inositol phosphate metabolism	−6.12	−5.03	−5.59	−5.19	−5.94
bme00960 (map00960)^*^	Alkaloid biosynthesis II	−6.24	−5.2	−6.06	−4.49	−7.29
bme00561	Glycerolipid metabolism	−6.52	−6.47	−5.85	−6.78	−5.49
bme00780	Biotin metabolism	−6.58	5.58	−8.34	−5.42	−6.92
bme00430	Taurine and hypotaurine metabolism	−6.77	−7.51	−5.9	−4.86	−7.34
bme00471	D-Glutamine and D-glutamate metabolism	−6.78	6.63	−6.03	−5.26	−6.85
bme00520	Nucleotide sugars metabolism	−6.78	−8.13	−9.91	−8.15	−7.92
bme00910	Nitrogen metabolism	−6.93	−7.05	−8.95	−4.62	−8.09
bme00120 (map00120)^*^	Bile acid biosynthesis	−7	−7.7	−6.81	−4.94	−6.98
bme00791 (map00791)^*^	Atrazine degradation	−7.07	−8.29	−8.65	−8.67	−5.95
bme00623 (map00623)^*^	2,4-Dichlorobenzoate degradation	−7.17	8.12	−7.34	−5.59	−8.6
bme00440	Aminophosphonate metabolism	−7.32	−7.74	−7.47	−8.84	−8.42
bme00362	Benzoate degradation via hydroxylation	−7.39	−6.05	−6.55	−7.82	−6.72
bme00930	Caprolactam degradation	−7.59	−8.47	−7.83	−6.26	−7.4
bme00626	Naphthalene and anthracene degradation	−7.67	−8.74	−7.96	−9.4	−9.68
bme00401	Novobiocin biosynthesis	−7.7	−9.01	−6.93	−6.7	−8.07
bme00564	Glycerophospholipid metabolism	−7.8	6.54	7.27	4.05	7.56
bme00750	Vitamin B6 metabolism	−7.87	−7.82	6.75	−6.35	−6.14
bme00363 (map00363)^*^	Bisphenol A degradation	−7.96	−8.65	−9.7	−4.4	−8.24
bme00903	Limonene and pinene degradation	−8.03	−9	−8.32	−4.84	−7.22
bme00260	Glycine, serine and threonine metabolism	−8.13	8.1	8.28	6.85	8.88
bme00980 (map00980)^*^	Metabolism of xenobiotics by cytochrome P450	−8.17	−6.12	−5.34	−5.38	−7.51
bme00380	Tryptophan metabolism	−8.21	−8.52	−6.83	−8.29	−7.72
bme00340	Histidine metabolism	−8.22	−7.84	−9.8	−6.14	−7.31
bme00053	Ascorbate and aldarate metabolism	−8.26	−9.15	−7.21	−4.6	−5.25
bme00624	1- and 2-Methylnaphthalene degradation	−8.35	8	9.52	4.59	9.26
bme00982 (map00982)^*^	Drug metabolism—cytochrome P450	−8.38	−6	−4.92	−4.92	−7.29
bme00410	Beta-Alanine metabolism	−8.46	−10.2	−6.21	−8.3	−6.83
bme00360	Phenylalanine metabolism	−8.47	6.77	7.76	3.73	8.95
bme00350	Tyrosine metabolism	−8.62	−9.09	−11.2	−5.31	−9.84
bme00310	Lysine degradation	−8.7	−8.34	−7.4	−7.87	−6.92
bme03430 (map03430)^*^	Mismatch repair	−8.7	−8.39	−7.72	−7.25	−6.91
bme00220	Urea cycle and metabolism of amino groups	−8.78	−8.52	−6.88	−6.64	−8.13
bme01040	Biosynthesis of unsaturated fatty acids	−9.05	−6.42	−6.49	−6.13	−7.2
bme00071	Fatty acid metabolism	−9.35	−9.94	−6.37	−8.2	−6.77
bme00281	Geraniol degradation	−9.41	−8.07	−7.56	−8.65	−9.49
bme00650	Butanoate metabolism	−9.89	−8.32	−7.93	−6.51	−8.96
bme00280	Valine, leucine and isoleucine degradation	−10.19	−7.17	−9.32	−5.61	−8.64
bme00040	Pentose and glucuronate interconversions	−10.28	−8.56	−9.77	8.41	−6.75
bme00640	Propanoate metabolism	−10.89	−7.64	−9.42	−6	−8.92

* *Indicates that current KEGG Database only includes the ‘map’ reference pathway*.

** *Indicates the “bme” pathway was combined with another pathway in the current KEGG database*.

The pathways are organized by category and then sorted by activated or repressed states on the T15 minute p.i. column. Pathway scoring employed the in vivo gene expression for the experimental treatment condition and the in vitro gene expression from the log growth phase of the inoculum as the control condition. The “bme” pathway molecular interactions were originally based on the 2009 version of Kyoto Encyclopedia of Genes and Genomes (KEGG) database and their KEGG pathway name designators (Kanehisa et al., 2017). Some pathway names indicated with a ^*^ or ^**^ required updating to be consistent with the current online KEGG database.

The TCRSs are signal transduction mechanisms that allow microorganisms to sense and respond to changes in environmental conditions. Bioinformatic analysis of *Brucella* genomes has identified 15 predicted *bona fide* TCRS pairs (Lavin et al., 2010). Several of these systems have been characterized in *Brucella* species, such as BvrSR (Sola-Landa et al., 1998), FeuQP (Dorrell et al., 1998), NtrBC (Dorrell et al., 1999), NtrXY (Foulongne et al., 2000; Carrica et al., 2012), PrlSR (Mirabella et al., 2012), the flagellar master regulator FtcR (Leonard et al., 2007), the blue-light-activated LOV HKs (Swartz et al., 2007), RegA (Abdou et al., 2017), and CenR (Zhang et al., 2009). Other TCRSs have been identified by transpositional mutagenesis during global screening for virulence factors (Lestrate et al., 2000; Wu et al., 2006) but remain uncharacterized. Our pathway analysis revealed that the TCRSs were initially repressed at 15 min. p.i. and were then activated for the remaining time points (Table 1) providing evidence that *in vivo Brucella* sense and actively regulate their metabolism through the transition to intracellular lifestyle. Changes in expression between 15 min to 30 min p.i. by the TCRSs genes *dctM, glnG, glnL, phoB, phoQ, citE*, and *divJ* resulted in the TCRSs pathway transitioning from a repressed state to an activated state with the exception of *aceA* which was activated early and then repressed. These genes went from a strongly repressed state to an insignificant expressed state. Contrarily, other *Brucella* pathways involved in virulence such as “ABC transporters” and “T4SS system” were continuously repressed suggesting a silencing strategy to avoid stimulation of the host's innate immune response very early in the infection process (Table 1). The highly repressed genes associated with the ABC transporters repression included BMEII0196, BMDII0861, PBMII0120, BMEI1138, *proW, ybbP, pstB, potB, afuB, rbsC* and several others. The T4SS system repression was induced by the repressed genes *virB4, virB5, virB6*, and *virB9* (Table S2). *In vitro* studies have demonstrated that T4SS is not required for cellular invasion, and its expression begins 15 min after phagocytosis and maximizes at 5 h p.i. (Sieira et al., 2004). It has been shown to be indispensable for intracellular survival of *Brucella* (O'Callaghan et al., 1999; Sieira et al., 2000; Delrue et al., 2005). Under our *in vivo* experimental conditions, expression of genes from the *virB* operon was repressed as was confirmed by qRT-PCR [Figure S1: BMEII0033 (virB9)]. In addition, the transcriptional regulator *vjbR* (BMEII1116) that positively regulates the expression of *B. melitensis virB* operon (Delrue et al., 2005) was not differentially expressed in our microarray results (Table S2). These data show that the T4SS was repressed during the first 4 h p.i. of *in vivo* infection. Collectively, our results, in addition to those reported earlier (Roux et al., 2007; den Hartigh et al., 2008) that failed to detect differences in the number of *B. abortus* and *B. melitensis* WT and *virB* mutant recovered from mice spleens in the first 3 days p.i., suggest that the *virB* operon may not play a major role in the initial *in vivo Brucella* pathogenesis. There are likely *in vivo* environmental signals that modulate the expression of the *virB* operon differently than reported in *in vitro* systems of infection. Identification of the host molecule targets of the T4SS will help characterize its expression based on the host cellular response discussed further in the next section “Systems biology *in vivo* interactome modeling results.”

### Systems biology *in vivo* interactome modeling results

The simultaneous collection of host and pathogen gene expression data of the bovine host ileal loop infected with *B. melitensis* WT (Rossetti et al., 2013), provided us with a unique opportunity to examine temporal host pathway perturbations concurrent with those of the pathogen. A computational approach based on DBN machine learning was employed to infer protein–protein interactions (PPIs) and to create a novel *in silico* host-pathogen interactome model (File S1). To identify plausible PPIs, a specialized application was developed to implement algorithms that integrate multiple sources of PBK such as from KEGG, BIOCARTA, NCBI, PIBASE, and *Brucella* proteomic analyses (Delvecchio et al., 2002; Wagner et al., 2002; Connolly et al., 2006; Mol et al., 2016), into the inference of host-pathogen protein interactions. Such interactions aid in the identification of mechanisms of host invasion and evasion through manipulation of the host's immune response system. Our application employed Bayesian networks (BNs) (Friedman et al., 2000; Hartemink et al., 2001) that were expanded by others to include PBK (Imoto et al., 2004; Werhli and Husmeier, 2007). We employed methods similar to Werhli for learning PPIs from expression data and PBK (Werhli and Husmeier, 2007). We adopted three algorithmic methods for the identification of candidate interaction points for use in network learning between the host and pathogen from *in vivo* gene expression data. Through either: (1) protein binding domain, (2) sequence similarity, or (3) Gene Ontology-based functional algorithms of pathogen gene expression with the host gene perturbations, we identified potential *B. melitensis* interactions with host pathways.

The PPI analysis resulted in identifying the virB gene that encodes the T4SS proteins to have plausible interaction with a number of genes in the host's immune response pathways (Table 2 and Table S8). For example, the significantly perturbed *virB11* gene has a high protein domain binding prediction with a negative correlation with the host gene/protein PIK3R2 expression. PIK3R2 is a key regulatory protein in several key pathways including: mTOR signaling, T-cell and B-cell receptor, Integrin-mediated cell adhesion, regulation of actin cytoskeleton, apoptosis, and Toll-like receptor. The host gene PIK3R2 remained activated for all time points p.i. Interestingly, in our host pathway analysis of “Regulation of Actin Cytoskeleton,” the genes ABI2, PPN1, and ARPC5, down-stream from PIK3R2, were repressed suggesting a mechanism of host pathway disruption or highjacking. The VirB11 also had a strong protein domain binding prediction with negative correlation with the host genes MAPK8IP1/2 of the MAPK signaling pathway. The MAPK8IP1/2 host genes were strongly repressed 15 min p.i. and insignificantly expressed thereafter. Down-stream of MAPK8IP1/2, the transcription factor JUND and nuclear factor of activated T-cells, cytoplasmic 3, NFATC2 are both strongly repressed.

**Table 2:** Interactome model predicted protein–protein interactions (PPIs) between bovine host and *B. melitensis* pathogen.

***B. melitensis* gene**	***B. melitensis* gene description**	**Normalized correlation weight**	**Bovine host gene**	**Bovine Gene Description**	**Host gene significantly perturbed**	**Prediction type**	**Perturbed Host Pathways**
MotB BMEI0324 (BME_RS01570)	Chemotaxis MotB protein	0.268	*JUN*	jun oncogene	Yes	PD	Toll-like receptor, MAPK, Epithelial cell signaling, GnRH signaling, ErbB signaling, Wnt signaling, BRC signaling, B cell receptor, T cell receptor
MotB BMEI0324 (BME_RS01570)		−0.221	*ROCK2*	Rho-associated, coiled-coil containing protein kinase 2	Yes	PD	Regulation of actin cytoskeleton, Axon guidance, Integrin-mediated cell adhesion, TGF-beta signaling, CCR@ signaling, Wnt signaling
BMEI0717 (BME_RS03560)	22 kDa OMP precursor	0.208	*BAD*	BCL2-antagonist of cell death	Yes	BSS	Trefiol Factors Mucosal Healing, VEGF signaling, Apoptosis
BMEI0717 (BME_RS03560)		0.188	*MAP2K3*	Mitogen-activated protein kinase kinase 3	Yes	BSS	MAPK, GnRH, Toll-like receptor, Fc epsilon RI signaling, Integrin-mediated cell adhesion
BMEI0890 (BME_RS04435, tgt)	tRNA guanosine transglycosylase	0.159	*RAP1A*	RAP1A, member of RAS oncogene family	No	BSS	MAPK, Integrin-mediated cell adhesion, Leukocyte transendothelial migration
BMEI1077 (BME_RS05395)	Immunogenic membrane protein YajC	−0.206	*NRAS*	Neuroblastoma RAS viral (v-ras) oncogene homolog	Yes	PD	ErbB signaling, Regulation of actin cytoskeleton, Natural killer cell mediated cytotoxicity, Tight junction, Fc epsilon RI signaling, T cell receptor signaling, GnRH signaling
BMEI1077 (BME_RS05395)		−0.225	*CBL*	Cas-Br-M (murine) ecotropic retroviral tranforming sequence	No	PD	Jak-Stat signaling, ErbB signaling, Insulin signaling, T cell receptor signaling
BMEI1077 (BME_RS05395)		−0.238	*RHOA*	ras homolog gene family, member A	Yes	PD	Trefoil factors, Tight junction, TGF-beta signaling, Wnt signaling, Adherens junction, Integrin-mediated cell adhesion, etc.
BMEI1086 (BME_RS05450)	Segregation and condensation protein A	−0.214	*CRKL*	v-crk sarcoma virus CT10 oncogene homolog	No	BSS	Regulation of actin cyctoskeleton, Insulin signaling, ErbB signaling, MAPK
BMEI1582 (BME_RS07890, narL)	Two-component system nitrate/nitrite response regulator	0.145	*MKNK1*	MAP kinase interacting serine/threonine kinase 1	Yes	BSS	MAPK, Insulin signaling
BMEI1751 (BME_RS08690)	LuxR family transcriptional regulator	−0.102	*IRF3*	Interferon regulatory factor 3	No	BSS	Toll-like receptor signaling
BMEI1846 (BME_RS09140)	Response regulator receiver protein ExsF	0.202	*FLNA*	Fliamin A, alpha (Actin binding protein 280)	Yes	BSS	MAPK
BMEI1846 (BME_RS09140)		−0.157	*CRK*	v-crk sarcoma virus CT10 oncogene homolog	Yes	BSS	Regulation of actin cytoskeleton, Insulin signaling, ErbB signaling, MAPK, Integrin-mediated cell adhesion
BtaE (BMEI1873)	trimeric autotransporter adhesin	−0.018	NFKBIA	nuclear factor of kappa light polypeptide gene enhancer in B-cells inhibitor, alpha	No	PD	CD40L Signaling, Apoptosis, Toll-like receptor signaling, Adipocytokine signaling, Epithelial cell signaling in Helicobacter pylori infection, Chronic myeloid leukemia, Prostate cancer, T cell receptor signaling with Antigen Processing, B cell receptor signaling, T cell receptor signaling
BtaE (BMEI1873)		0.035	NFATC2	nuclear factor of activated T-cells, cytoplasmic, calcineurin-dependent 2	No	PD	Wnt signaling, VEGF signaling, T cell receptor signaling with Antigen Processing, T cell receptor signaling, MAPK signaling, Calcium signaling, Natural killer cell mediated cytotoxicity, Axon guidance
BtaE		−0.037	MAPK8IP1	mitogen-activated protein kinase 8 interacting protein 1	No		MAPK signaling
BtaE		0.014	RRAS2	related RAS viral (r-ras) oncogene homolog 2	No	BSS	Regulation of actin cytoskeleton, MAPK signaling, Insulin signaling, Axon guidance, Long-term depression, T cell receptor signaling, Natural killer cell mediated cytotoxicity, Gap junction, Tight junction, B cell receptor signaling, Long-term potentiation
BtaE		0.03	RAC3	ras-related C3 botulinum toxin substrate 3 (rho family, small GTP binding protein Rac3)	No	BSS	Integrin-mediated cell adhesion, Colorectal cancer, Pancreatic cancer, VEGF signaling, MAPK signaling, Fc epsilon RI signaling, Toll-like receptor signaling, Regulation of actin cytoskeleton, Adherens junction, Wnt signaling, B cell receptor signaling, Axon guidance
BtaE		−0.028	CCND1	cyclin D1	No	BSS	Colorectal cancer, Wnt signaling, Acute myeloid leukemia, Thyroid cancer, Prostate cancer, Endometrial cancer, Non-small cell lung cancer, Chronic myeloid leukemia, Bladder cancer, Glioma, Pancreatic cancer, Melanoma, Small cell lung cancer, Jak-STAT signaling, Cell cycle
BMEI1872 (BME_RS09280)	Cell surface protein	−0.153	*JUND*	jun D proto-oncogene	Yes	BSS	MAPK
BMEII0035 (BME_RS10365, virB11)	P-type DNA transfer ATPase VirB11	−0.134	*PIK3R2*	Phosphoinositide 3 kinase, regulatory subunit 2	Yes	BSS	T cell receptor, mTOR signaling, VEGF signaling, ErbB signaling, Toll-like receptor, Apoptosis, Jak-STAT signaling, Phosphatidylinositol signaling, etc.
BMEII0035 (BME_RS10365, virB11)		−0.168	*MAPK8IP1*	Mitogen-activated protein kinase 8 interacting protein 1	Yes	PD	MAPK
BMEII0926 (BME_RS14720, minD)	Septum site-determining protein MinD	0.121	*RASGRP3*	RAS guanyl releasing protein 3	Yes	BSS	MAPK, B cell receptor signaling
BMEII0926 (BME_RS14720, minD)		0.102	*CBLB*	Cas-Br-M (murine) ecotropic retroviral tranforming sequence	Yes	BSS	T cell receptor signaling, Insulin signaling, ErbB signaling, Jak-STAT signaling
BMEII0951 (BME_RS14810, narH)	Nitrate reductase beta subunit	0.13	*CRK*	v-crk sarcoma virus CT10 oncogene homolog	Yes	BSS	Regulation of actin cytoskeleton, Insulin signaling, ErbB signaling, MAPK, Integrin-mediated cell adhesion
BMEII1085 (BME_RS15470, flgA)	Flagellar basal body P-ring biosynthesis protein FlgA	0.116	*CASP4*	Caspase 4, apoptosis-related cysteine peptidase	No	BSS	MAPK
BMEII1085 (BME_RS15470, flgA)		−0.105	*CASP3*	Caspase 3, apoptosis-related cysteine peptidase	Yes	BSS	Apoptosis, MAPK, Natural killer cell mediated cytotoxicity
BMEII1113 (BME_RS15605, fliG)	Flagellar motor switch protein FliG	−0.112	*MAP4K1*	Mitogen-activated protein kinase kinase kinase kinase 1	Yes	BSS	MAPK

*Higher evidence of possible interaction is inferred if the host gene is also significantly perturbed and correlated with the pathogen gene expression. Prediction type of “Protein Domain” (PD) means that the same binding domains exist between host-pathogen proteins. “Binding sequence similarity” (BSS) is achieved by finding similar sequence in the pathogen as a binding protein domain existing in the host protein. Correlation weights have been normalized so that comparisons between different pathways can be made. The pathways which contain the host PPI genes are listed for each PPI pair. Both the in vivo pathogen and host gene expressions were employed in training the models for learning the PPIs*.

*Brucella* flagellum is a virulence factor transiently expressed during vegetative growth and required for persistent infection, but not for internalization *in vivo* (Fretin et al., 2005). In agreement, our results during the first 4 h of infection showed a repression of the three flagellum-encoded loci (BMEII0150-0168, BMEII1080-1089, and BMEII1105-1114) (Table S3), with corresponding repression of the “flagellar assembly” pathway (Table 1), and the cell components “bacterial-type flagellum hook” and “bacterial-type flagellum” (Table S5) in tissue-associated *B. melitensis* compared with *in vitro*-grown cultures. In addition, *RopE1* sigma factor (BMEI0371), a flagellar repressor (Ferooz et al., 2011), was activated throughout the experiment (Table S3). We further examined potential interaction of the flagella-associated genes with the host. Three highly correlated PPIs were identified which included the flagella genes BMEI0324, BMEII1085 (*flgA*), and BMEII1113 (*fliG-2*). The ORF BMEI0324 had strong binding sequence similarity and positive correlation to the host expressed JUN (jun oncogene) which is part of the highly perturbed host pathways: Toll-like Receptor, ErbB Signaling, BRC Signaling, B-cell Signaling, T-cell Signaling, Epithelial Cell Signaling, WNT Signaling, and MAPK Signaling. The flagellum gene *flgA* also had strong binding sequence similarity and positive gene expression correlation to host *CASP2* gene while having a reversed (negative) correlation with the host activated *CASP3* gene. Interestingly, the lowly expressed *CASP2* is only associated with the highly perturbed MAPK signaling pathway, while *CASP3* has several pathway associations that include: Apoptosis, Epithelial Signaling, Natural Killer Cell, and MAPK Signaling. The third flagellum gene *fliG-2* had strong binding sequence similarity to the host *MAP4K1* gene. The highly activated *MAP4K1* is associated with only the host MAPK Signaling pathway. Such interactions may be novel virulence candidates that facilitate circumventing the host immune response. An example of interaction is the pathogen gene *flgA* with the host gene/protein Casp4/6/7/9 which are MAPK pathway genes. Accordingly, down-stream of Casp genes are the repressed *RAC1/2/3* genes of the Rho family of GTPases. *RAC1* has been implicated in various downstream cellular functions, including, but not limited to, cellular plasticity, migration and invasion, cellular adhesions, cell proliferation, and apoptosis.

Host cells identify specific pathogen-associated molecular pattern (PAMP) motifs present in the bacteria by pattern-recognition receptors (PRRs), such as Toll-like Receptors (TLRs). These receptors are key to establishing an important network between the innate and adaptive immune systems. TLR5 is the cellular receptor for extracellular flagellin, a major structural protein of Gram-negative flagella. Binding of flagellin to the extracellular domain of TLR5 rapidly induces a signal cascade that culminates in the production of proinflammatory mediators such as cytokines, chemokines, and costimulatory molecules (Honko and Mizel, 2005). Therefore, the absence of flagellum apparatus during extracellular life while inside the host suggests the *Brucella* strategy is to avoid triggering a host immune response and an initiation of a *Brucella* persistence mechanism (Terwagne et al., 2013). However, our previous analysis showed that TLR5 pathway is activated in *B. melitensis*-infected bovine Peyer's patches during the first hour p.i. (Rossetti et al., 2013), which may have been associated with remnants of flagella in the *in vitro*-growth culture media intraluminally inoculated. More detailed analysis of this pathway showed that down-stream of *TLR5* there were several strongly repressed genes including *PIK3C2B, PIK3R4, STAT1, AKT3, RAC3, IL6*, and *TICAM3*. This may suggest that the pathogen is manipulating important signaling processes by some other mechanism. PPI analysis indicated that virB genes have predicted interactions with *STAT1, PIK3C2B*, and *IL6* which may also be circumventing the TLR5 response to flagellin stimulation and preventing the host from mounting an effective immune response.

**Figure 2:**
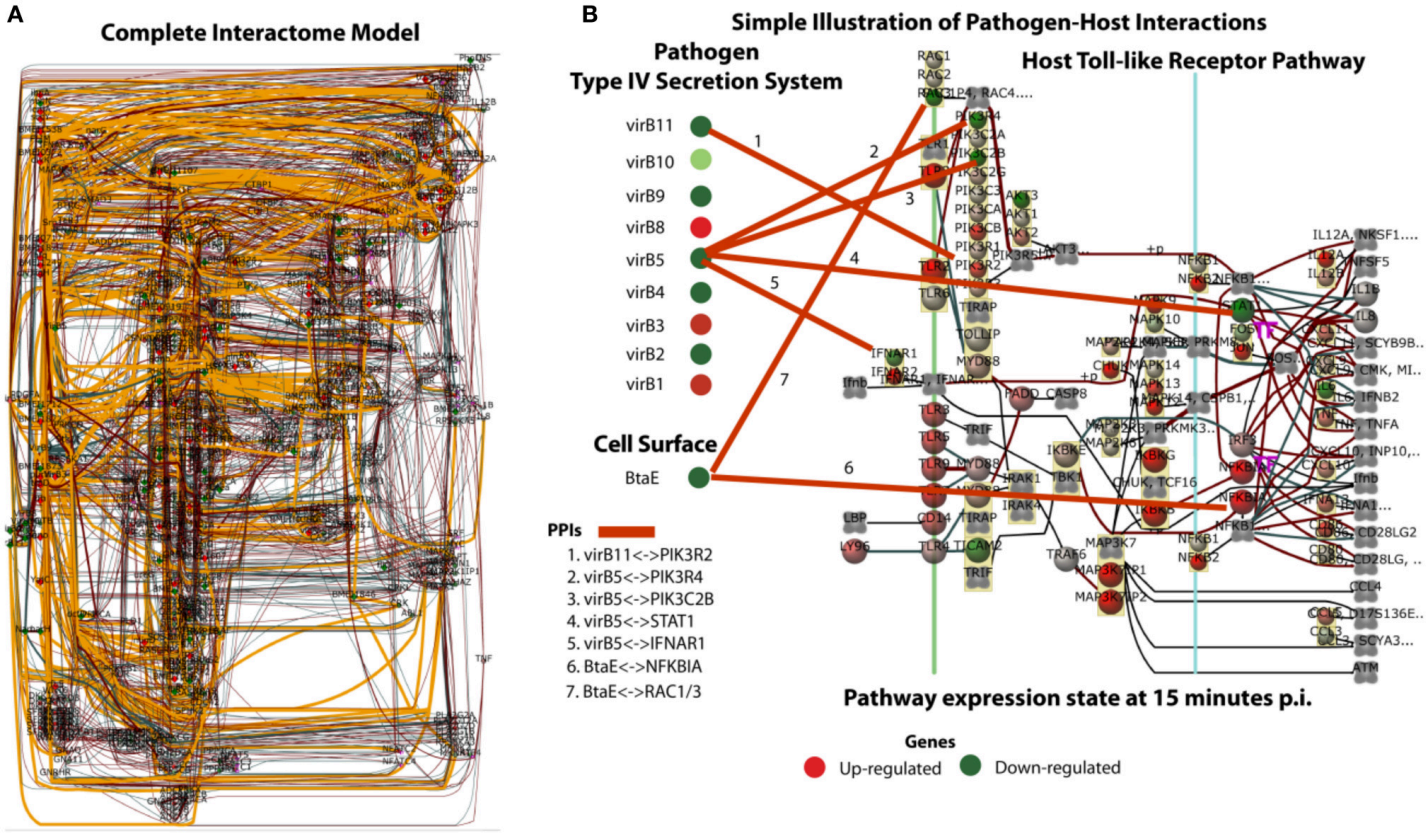
**(A)** The Bayesian network for host-pathogen interactome model for bovine Peyer's patch challenged with *B. melitensis*. **(B)** Simplification of the interactome to illustrate the points of interaction between pathogen's Type IV secretion system and the cell surface gene BtaE with the host's Toll-like receptor pathway. The arcs show the points of predictive interaction which could be possible mechanisms of disrupting the host's effective immune response *to B. melitensis*.

The complexity of a complete system-level host and *B. melitensis* interaction model (*G*_*interactome*_) is illustrated in Figure 2A. This Bayesian network model is comprised of approximately 528 nodes (genes) and 987 arcs that connect gene nodes (relationships). Of the 987 arcs, 101 arcs were learned for host-pathogen points of interaction which are highlighted by the orange colored arcs. The number of host genes were limited to a selected set of perturbed host pathways which included MAPK signaling, ErbB signaling, mTOR signaling, WNT signaling, VEGF signaling, Toll-like Receptor signaling, GnRH signaling, Tight junction, Phosphatidylinositol signaling, Notch signaling, Natural killer cell mediated cytotoxicity, and Apoptosis. The intent for using only perturbed pathways was to look for plausible points of pathogen interactions which could influence the hosts immune response. Although this model is visually complex, the model allows for the computational extraction of potentially important mechanisms of interaction. Of the 101 arcs, the prioritization of these interactions can be analyzed according to the most likely to least likely in the following order: “protein domain,” “sequence similarity,” “GO Functionality,” and “Correlated Data”. The creation of the interactome model employed only PPI relationships based on protein domain or sequence similarity. For example, Figure 2B illustrates the simplification for the interaction between the pathogen's Type IV secretion system and a known cell surface protein, BtaE (Ruiz-Ranwez et al., 2013b), with the host's Toll-like receptor pathway. This demonstrates how predictive information can be employed to interpret host-pathogen responses. The thicker orange arcs are the connections from the pathogen to the host. From this type of analysis, it is possible to understand the state of host's gene expression down-stream from the potential points of interaction/disruption in any of the pathways showing potential manipulation by the pathogen. Table 2 lists a selected subset of predicted interactions representing the pathogen-host pairs based solely on “protein domain” and “sequence similarity” prediction. A complete listing of predicted PPIs based on protein domain or sequence similarity is provided in Table S8. Note that our computational approach did predict interactions of the *B. suis* gene BtaE with several host proteins listed in Table 2. BtaE belongs to the type II (trimeric) autotransporter family and is an orthologue of *B. melitensis* BMEI1873. BtaE has been shown to have an active role in host cell adhesion and binding with components of the extracellular matrix such as fibronectin, collagen, and vitronectin (Ruiz-Ranwez et al., 2013b). The interactome model and its predicted PPI list is the analysis output to be employed for further *in vitro* validation and model refinements. The resulting *B. melitensis* PPI gene set may represent important new virulence factors with the potential to disrupt or hijack the host immune response. Table 2 also lists the perturbed host pathways in which the host gene PPI is associated, intentionally unfiltered conceptually for subcellular locations so that all PPI are presented. Little is known about the complete secretome of *B. melitensis* during *in vivo* host invasion and proliferation although the secretion systems and secretomes of *Brucella* were recently computationally analyzed which predicted 29 host-pathogen specific interactions between cattle and *B. abortus* and 36 host-pathogen interactions between sheep and *B. melitensis* proteins (Sankarasubramanian et al., 2016). The PPI computational approach employed evidence of host pathway perturbation and gene expression disruption as possible indicators of pathogen interaction. If there was plausible potential for a PPI based on binding domain or sequence similarity to known protein interactions between the pathogen and host protein, then it was included in the PPI in list (Table 2 and Table S8). The list of PPIs can be prioritized based on the normalized correlation weights. The larger the normalized weight indicates stronger likelihood of a relationship between the pathogen and host genes. Note that the normalized correlation weight is an output of structure learning and is employed in the acceptance or rejection of an arc (edge) in the final Bayesian network. Arc weight is dependent on the number of incoming arcs to a node and other factors and should not be confused as a true correlation measurement between two gene expression values.

It is thought that the unfiltered PPI predictions could, in future experiments, employ such information as normalized correlation weights and cellular localization to help prioritize the selection of which PPI to be experimentally validated. Further, it is proposed that the evidence driven computational approach (*in vivo* host-pathogen responses and machine learning) for predicting bacterial and host cell protein interactions will narrow the focus on likely PPI candidates and will greatly enhance our capacity to design hypothesis-driven experimental approaches to discover which *Brucella* proteins directly participate in host interactions.

In conclusion, the *in silico* interactome modeling offers informative insights leading toward new hypotheses regarding host-pathogen mechanisms of invasion and evasion. This modeling infers that *B. melitensis* has multiple points of host interaction that occur at the early stage post infection. A number of important innate immune response pathways appear to be potential targets of disruption by invading *B. melitensis*, such as Regulation of Actin Cytoskeleton, mTOR Signaling, MAPK, and Toll-like Receptor Signaling appear to be likely targets of pathogen manipulation that warrant further exploratory research and verification of PPIs. As we have reported and discussed here, identifying interactive host:pathogen PPIs is often the initial step to establish functional significance according to the principle of “guilty by association” (Schauer and Stingl, 2009) that may drive future research to a higher level of understanding of the molecular pathogenesis of brucellosis, thereby facilitating the design of novel immunotherapeutic drugs and vaccines.

## Ethics statement

This study was carried out in accordance with the recommendations of the Texas A&M University Institutional Animal Care and Research Advisory Committee. The protocol (AUP#2003-178) was approved by the Texas A&M University Institutional Animal Care and Research Advisory Committee.

## Author contributions

Conceived and designed the experiments: CR and LA. Performed the experiments: CR, SL, JN, TG, SK, and LA. Analyzed the data: CR, KD, and LA. Writing—original draft: CR, KD, and LA. Writing—review & editing: CR, KD, SL, JN, TG, SK, and LA.

### Conflict of interest statement

KD is a Chief Technology Officer in Seralogix, LLC. Seralogix is a bioinformatics research and services company commercializing computational systems biology software tools that are being sponsored by the National Institute of Allergy and Infectious Diseases and the National Human Genome Research Institute. KD participated in conducting certain genomic data processing involving pathway analyses and modeling that helped to provide a more system-level perspective of the host-pathogen interaction to the Texas A&M University researchers. Data were processed by KD utilizing Seralogix's proprietary computational pipeline for biological systems analysis. The relation between Seralogix and Texas A&M University, College of Veterinary Medicine and Biomedical Science is strictly on a collaborative (mutually beneficial) research basis with no financial arrangements, commitments or interests. KD's motivation is to see their computational tools produce results that contribute to the improved understanding of host response to pathogen invasions (an objective of his National Health Institute research grants). KD contributed to the interpretation of the analysis results provided to the Texas A&M University researchers. Seralogix has no ownership of the data, nor results produced by their tools. The other authors declare that the research was conducted in the absence of any commercial or financial relationships that could be construed as a potential conflict of interest.

## Supplementary material

Figure S1 Validation of *Brucella melitensis* microarray results by quantitative real time PCR. Six randomly selected *B. melitensis* ORFs that were consistently perturbed in microarray results in the first 4 h p.i. as compared to the inoculum as validated by quantitative RT-PCR. Fold-change was normalized to the expression of *B. melitensis* 16s rRNA and calculated using the ΔΔC_*t*_ method. All tested genes at every time point had expression altered in the same direction as microarray. Open bars represent fold-change by microarray analysis and black bars represent fold-change by qRT-PCR.

Table S1 Primers for Real Time PCR analysis of genes in *B. melitensis* samples.

Table S2 Bayesian z-score of *B. melitensis* genes in tissue-associated *B. melitensis* from 15 min to 4 h post-infection. Genes with *z*-score >|2.24| were considered differentially expressed. Positive numbers in the body of the table indicate activated genes and negative (−) numbers indicate repressed genes. The tissue-associated *B. melitensis* gene expression at every time point was compared to the gene expression of the inoculum (i.e., *in vitro*-grown cultures of *B. melitensis* at late-log phase of growth). Measured time points were 15 (T15), 30 (T30), 60 (T60), 120 (T120), and 240 (T240) min p.i.

Table S3 Core set of *B. melitensis* genes differentially expressed (>|2.24|) in at least four of five time points in the first 4 h post-infection of bovine Peyer's patch. Genes with *z*-score >|2.24| were considered differentially expressed. Positive numbers in the body of the table indicate activated genes, negative (−) numbers indicate repressed genes. The tissue-associated *B. melitensis* gene expression at every time point was compared to the gene expression of the inoculum (i.e. *in vitro*-grown cultures of *B. melitensis* at late-log phase of growth). Measured time points were 15 (T15), 30 (T30), 60 (T60), 120 (T120), and 240 (T240) min p.i.

Table S4 Significantly perturbed Biological Processes (BP) (Bayesian *z*-score >|2.24|) of tissue-associated *B. melitensis* during the first 4 h post-bovine Peyer's patch infection. Measured time points were 15 (T15), 30 (T30), 60 (T60), 120 (T120), and 240 (T240) min p.i. The Bayesian *z*-scores represent the degree of perturbation of the group of BP genes in tissue-associated *B. melitensis* versus the control. Positive z-scores represent activation of the BP (BP score is dominated by more activated genes), while the negative (−) *z*-score represents BP repression (BP score is dominated repressed genes).

Table S5 Significantly perturbed Cellular Components (CC) (Bayesian *z*-score >|2.24|) of tissue-associated *B. melitensis* during the first 4 h post-bovine Peyer's patch infection. Measured time points were 15 (T15), 30 (T30), 60 (T60), 120 (T120), and 240 (T240) minp.i. The Bayesian *z*-scores represent the degree of perturbation of the group of CC genes in tissue-associated *B. melitensis* versus the control. Positive z-scores represent activation of the CC (CC score is dominated by activated regulated genes), while the negative (−) *z*-score represents CC repression (CC score is dominated by repressed genes).

Table S6 Significantly perturbed Molecular Functions (MF) (Bayesian *z*-score >|2.24|) of tissue-associated *B. melitensis* during the first 4 h post-bovine Peyer's patch infection. Measured time points were 15 (T15), 30 (T30), 60 (T60), 120 (T120), and 240 (T240) min p.i. The Bayesian *z*-scores represent the degree of perturbation of the group of MF genes in tissue-associated *B. melitensis* versus the control. Positive *z*-scores represent activation of the MF (MF score is dominated by more activated genes), while the negative (−) -score represents MF repression (MF score is dominated by repressed genes).

Table S7 Dynamic Bayesian pathway analysis scores along with the associated individual genes and their Bayesian Scores by time point post-inoculation of tissue-associated *B. melitensis* during the first 4 h post-bovine Peyer's patch infection. Measured time points were 15 (T15), 30 (T30), 60 (T60), 120 (T120), and 240 (T240) min p.i. Positive *z*-scores represent activation of genes while negative (−) -scores represent repressed genes).

Table S8 Comprehensive list of predicted host:pathogen tissue-associated *B. melitensis*:bovine protein–protein interactions (PPI). This list includes only those PPIs which were learned by either known protein domain binding or sequence similarity to known binding domains employing our Bayesian methods for PPI prediction.

File S1 Host-Pathogen Protein-Protein Interaction (PPIs) Prediction.
