# Supplementary figures and images for: Systems Biology Analysis of Temporal In vivo Brucella melitensis and Bovine Transcriptomes Predicts host:Pathogen Protein–Protein Interactions
Source: Front Microbiol. 2017 Jul 27;8:1275. doi: 10.3389/fmicb.2017.01275 (PMC5529337; doi:10.3389/fmicb.2017.01275)

**S1 Figure. qRT-PCR Validation of Microarray Gene Expression Data.**

**BMEI0475**

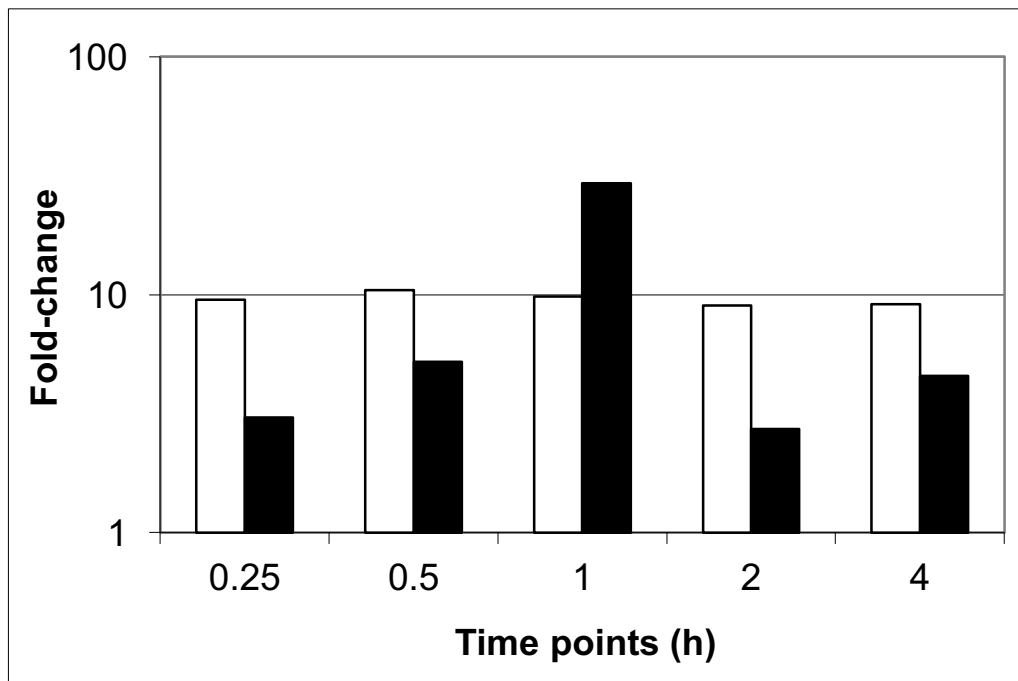

**BMEI0526**

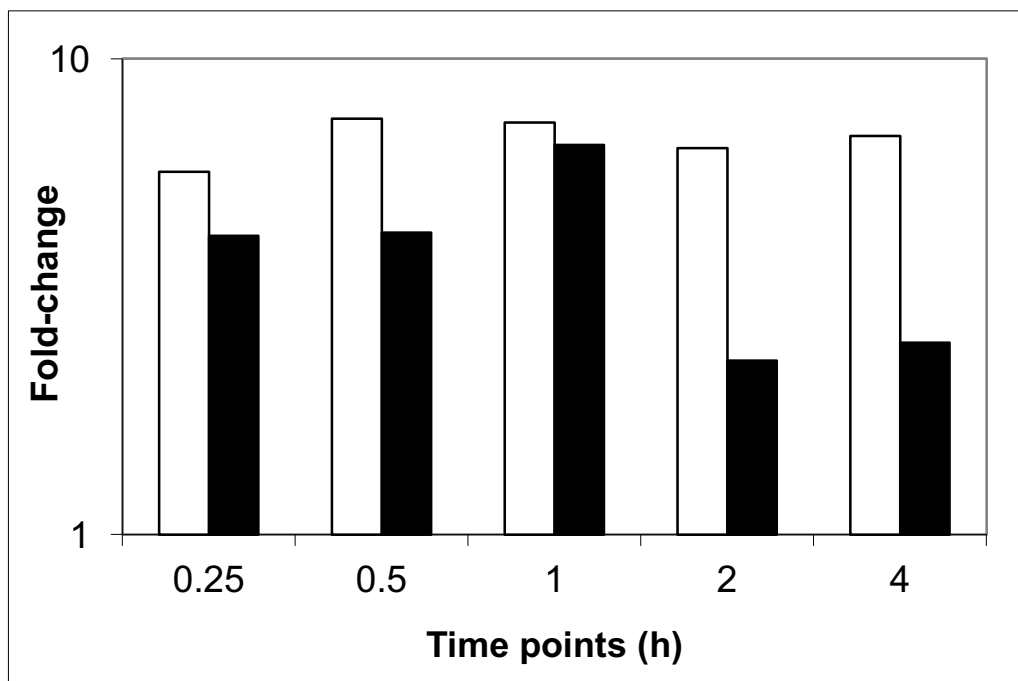

**BMEI1384**

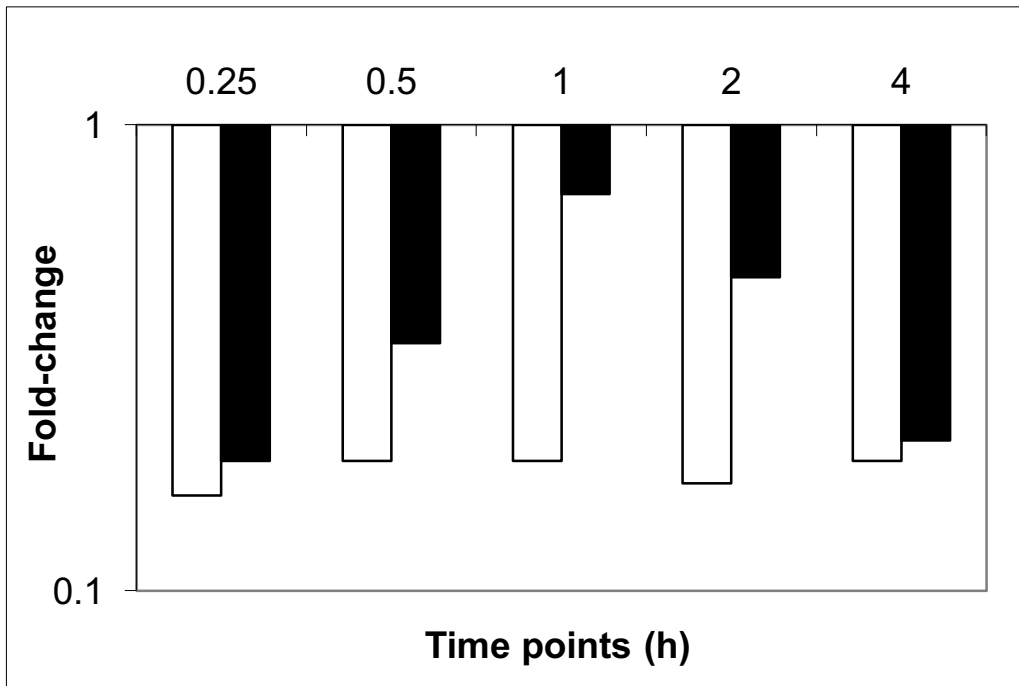

**BMEI1440**

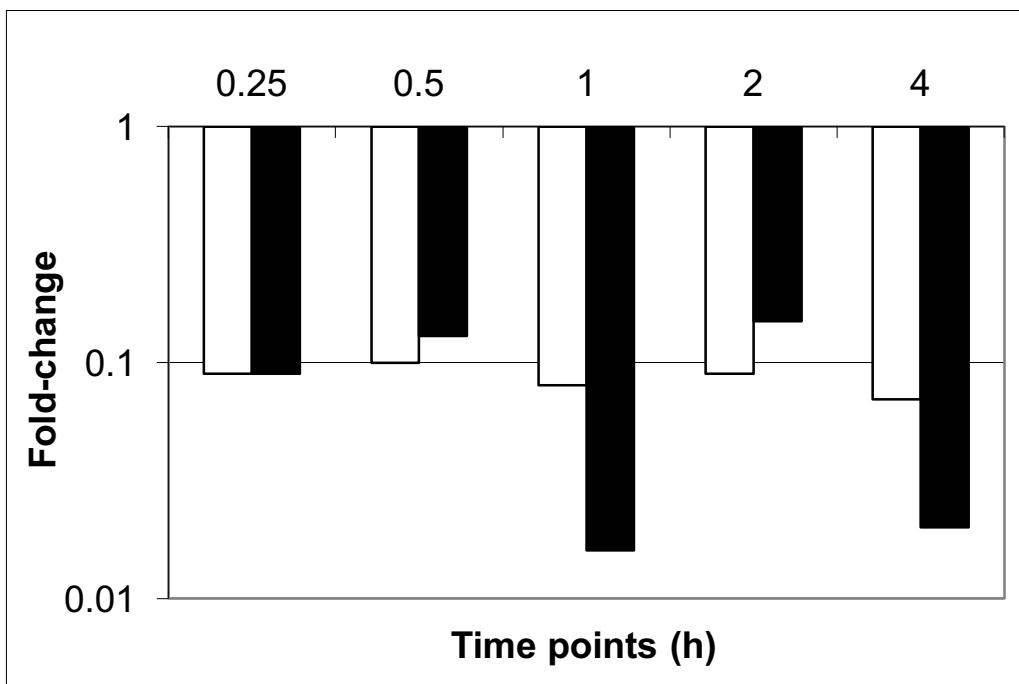

**BMEI1798**

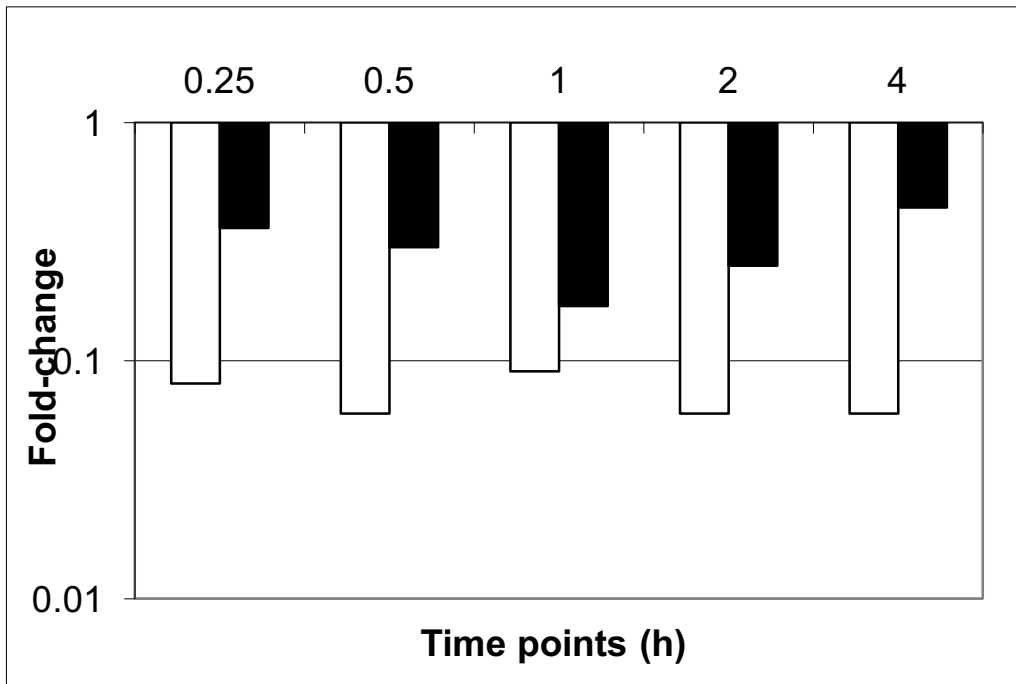

**BMEI10033**

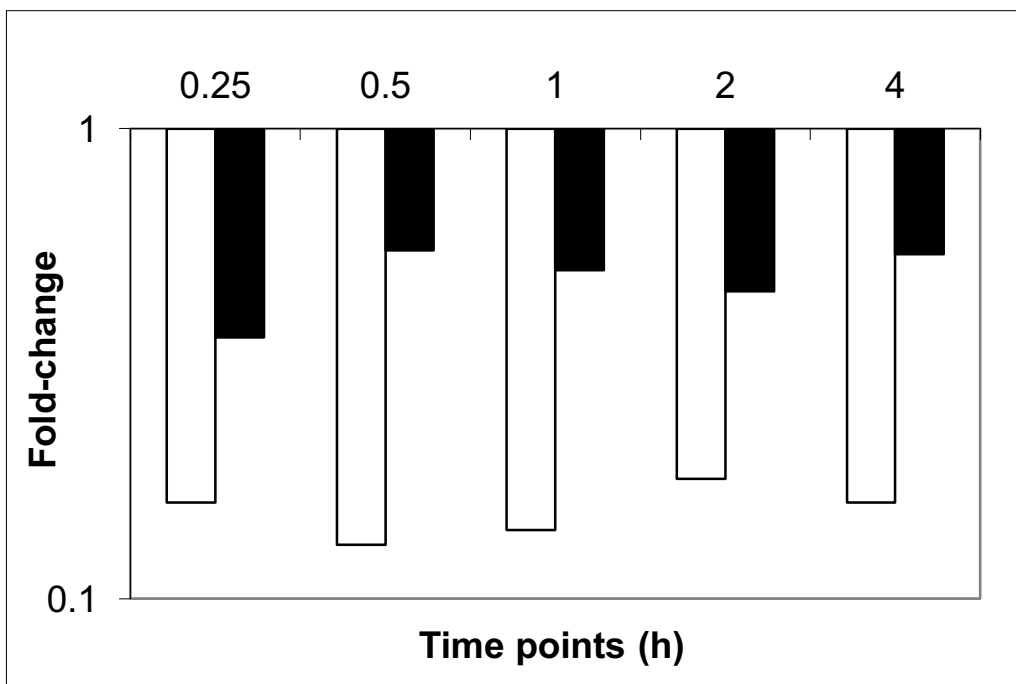

Supplement: Figure S1 — Validation of Brucella melitensis microarray results by quantitative real time PCR. Six randomly selected B. melitensis ORFs that were consistently perturbed in microarray results in the first 4 h p.i. as compared to the inoculum as validated by quantitative RT-PCR. Fold-change was normalized to the expression of B. melitensis 16s rRNA and calculated using the ΔΔCt method. All tested genes at every time point had expression altered in the same direction as microarray. Open bars represent fold-change by microarray analysis and black bars represent fold-change by qRT-PCR. [file Image1.PDF]
