# Supplementary material for: Systems Biology Analysis of Temporal In vivo Brucella melitensis and Bovine Transcriptomes Predicts host:Pathogen Protein–Protein Interactions
Source: Front Microbiol. 2017 Jul 27;8:1275. doi: 10.3389/fmicb.2017.01275 (PMC5529337; doi:10.3389/fmicb.2017.01275)
Supplement: Table S1 — Primers for Real Time PCR analysis of genes in B. melitensis samples. [file Table1.PDF]

S1 Table. Primers for Real Time – PCR analysis of genes in *Brucella melitensis* samples.

LOCUS ID	GENE NAME	FORWARD PRIMERS (5'-3')	REVERSE PRIMERS (5'-3')
BMEI0475 (BME_RS02375)	Ribosomal protein P2	GCTGCAGCGGCTAATAATGG	CGGTCAAAAGCGAATGGATATAA
BMEI0526 (BME_RS02635)	Carbamoyl-phosphate synthase small subunit	CGGTCAGAAGGCGCAGAATA	CTCGCCAAGGATGTCACCAT
BMEI1384 (BME_RS06965)	Transcriptional regulator, AraC family	CGCAGTTCACCAAGGCATT	GCGTGTTCAGAGGCGATCTT
BMEI1440 (BME_RS07205)	DSBA Oxidoreductase	CGAAATTGGCCGGTTTTACA	CCCGACATCTCCTCAAACGA
BMEI1798 (BME_RS08910)	RNA methyltransferase	CATGGGCTCGGTCTTTTCC	TGTTCATTGCCCATTATCAGGAT
BMEII0033 (BME_RS10355)	P-type conjugative transfer protein VirB9	CGATGCAGGTCGGCACTAAT	TGGCTGTTCACGATGCTTTC
BMEII0529 (BME_RS12775; *rrnA*)	16S rRNA	CCTTACGGGCTGGGCTACA	TGATCCGCGATTACTAGCGATT
