# Supplementary material for: Systems Biology Analysis of Temporal In vivo Brucella melitensis and Bovine Transcriptomes Predicts host:Pathogen Protein–Protein Interactions
Source: Front Microbiol. 2017 Jul 27;8:1275. doi: 10.3389/fmicb.2017.01275 (PMC5529337; doi:10.3389/fmicb.2017.01275)
Supplement: File S1 — Host-Pathogen Protein-Protein Interaction (PPIs) Prediction. [file Presentation1.PDF]

Supplemental File 1.

Host-Pathogen Protein-Protein Interaction (PPIs) Prediction.

A specialized application was developed to implement algorithms that integrate multiple sources of prior biological knowledge into the inference of host-pathogen PPIs. Bayesian networks (**BN**s) have been introduced to the problem of reconstructing gene regulatory networks from expression data by Friedman *et al.* [1] and Hartemink *et al.* [2] and expanded by others to include PBK such as by Imoto *et al.* [3] and Werhli and Husmeier [4]. We employed methods similar to Werhli and Husmeier (2007). Our networks of interactions are defined by a **BN** where the structure G of the **BN** is a directed acyclic graph (DAG) representing a linear Gaussian joint probability defined by $P(n_i) = \prod_{i=1}^{M} P(n_i | n_{p_i[G]})$ $\langle \boldsymbol{Eq\ 1} \rangle$, where M is the number of gene/protein nodes n_i in graph G and $n_{p_i[G]}$ represents the parents of node n_i. Currently, we adopted three algorithmic methods for the identification of candidate interaction points for use in network learning between the host and the pathogen from *in vivo* gene expression data. This network is learned by maximizing the likelihood $P(G|D) \propto P(D|G)P_{prior}(G)$ $\langle \mathbf{Eq\ 2} \rangle$, where D is the co-expressed gene data and $P_{prior}(G)$ is the prior distribution over possible network structures. Note that gene candidates for inclusion in our interaction prediction process were selected based on interpretation of pathway and GO analyses conducted by our Dynamic Bayesian Network methodology referenced in the main text. The *B. melitensis* (BME) gene transcriptome was employed and ≈600 bovine host genes selected from 12 perturbed and immune response relevant pathways and 10 GO terms to form gene sets representing two "unconnected" system models for starting the "interactome model" network learning process. The starting baseline **BN** structure G_{intra} for use in our network structure learning algorithm was derived from these host and pathogen system models by converting KEGG, BIOCARTA or NCBI interaction pathway entries e into their associated individual gene/protein nodes n_i. This process results in a baseline structure in which host and pathogen do not interact (at this point), hence the designation here as G_{intra}(i.e., all interactions are intra-host or intra-pathogen). Note that the structure learning algorithm is applied only to the interface between host and pathogen (which is yet to be derived), with the intra-host

and intra-pathogen structure held fixed. A key strength of Bayesian statistical methods is the ability to incorporate so-called "prior" information into estimates of likelihood. The purpose of inclusion of priors in structure learning is to effectively limit the number of structures considered to be the most biologically relevant. This Bayesian prior information is here specified in the form of an *a priori* most likely structure G_{ml} and a rate w of decline in prior likelihood $P_{prior}(G)$ as the number of arcs from G_{ml} missing from the structure G increases given by: $P_{prior}(G) = N\exp\left(-w \sum_{i,j} \max\left((G_{ml})_{ij} - G_{ij}, 0\right)\right)$ $\langle \textbf{Eq 3} \rangle$, where $G_{ij} = 1$ if an arc exists in G from node i to node j and $G_{ij} = 0$ otherwise, with $(G_{ml})_{ij}$ similarly defined, and Na normalization factor. The rate w (i.e, "prior weight") controls the degree of influence the prior structure exerts on the outcome of the structure learning. The methods we found most successful for construction of the prior structure G_{ml} were: 1) a sequence-similarity interaction transference; 2) structural protein domain-based algorithm; and 3) a functional gene-ontology-based algorithm.

1. Sequence-similarity Interaction Transference. The pathogen (BME) protein sequences were aligned against the bovine host proteins using the Smith-Waterman algorithm to determine sequence similarity using the program ssearch36 from FASTA [5,6]. Pathogen-host protein sequence matches were considered significant at E-values of 1.0 and below and limited to the 5 most significant. For each accepted pathogen-host pair match *P-H1*, all protein interaction partners *H2* of the host protein *H1* were taken as potential interaction partners for the protein *P*. The NCBI interaction database [7] was the source for known host side interaction partners. In the BME-Host analysis, there were 348 potential host-pathogen PPIs identified via this sequence similarity interaction transference procedure.

2. Protein-domain Interaction Transference. The HMMER (hmmscan) software was used to analyze protein sequences for the presence of Pfam [8] domains. By adjusting the match threshold criterion, domain models may be required to be of greater or lesser similarity to be matched to protein subsequences. This allows for the identification of more potential PPIs at the cost of decreased certainty (which can be reflected by adjusting the prior weight *w*). PPIs were then predicted on the basis of known

domain-domain interactions found in the PIBASE [9] database. This protein-domain-based interaction prediction method (for E-value threshold of 1.0) yielded 68 potential host-pathogen interactions.

3. Gene Ontology-based Functional Algorithm. We applied the gene ontology-based algorithm developed by Schlicker *et al.* [10] to measure functional similarity to predict host-pathogen PPIs. The metric quantifies the similarity of GO group annotations for any pair of genes/proteins from 0 and 1 and is used to predict possible interacting protein pairs in two ways. First, if functional similarity of two proteins has been observed to correlate weakly with interaction of the pair, it can be used as a direct predictor of potential interactions [11, 12]. We applied a GO similarity score threshold of 0.8, as suggested by Schlicker *et al.* (2006) to denote "high functional similarity" [10]. The second method transfers the known interaction partners of a host protein to any pathogenic proteins with which it shares high functional similarity (threshold of 0.8). These two methods yielded 295 potential host-pathogen PPIs.

These three PPI prediction methods generated the prior max-likelihood structure G_{ml} (and thus $P_{prior}(G)$, see Eq. 3) and the supplemental interactions G_{go}), which together composed the set of potential interactions at the interface of the pathogen and host systems. These potential interactions were then fed into the network structure learning algorithm described next for inclusion of host and pathogen expression data.

Bayesian Host-Pathogen Network Interaction Structure Learning Approach.

Key components of a structure learning algorithm include searching for "good" structures and scoring these structures. Since the number of model structures is large (super-exponential in number of nodes), a search method is needed to decide which structures to score. Even with few nodes, there are too many possible networks to exhaustively score each one. Hence, our method employs a prior structure $\mathrm{P_{prior}(G)}$ from above to initialize learning with biologically relevant structures and utilize actual time-course co-expressed gene and other "omic" data from pathogen and host to search for a set of structures in which the data best fits. Gene/protein data are often sparse and noisy such that the posterior probability is no

longer a sharp distribution over structure space. Accordingly, an ensemble of equilibrating structures is preferred to a single most-likely structure. Following a similar direction as Werhli and Husmeier [4], we employ a Markov Chain Monte Carlo (**MCMC**) simulation method [13]. That is, given a network structure G_{old}, a new network structure G_{new} is proposed based on asampling proposal distribution$Q(G_{old}|G_{new})$;$G_{new}$is subsequently accepted or rejected according to the Metropolis-Hastings acceptance probability: $A=\min\left\{\frac{P(D|G_{new})P_{prior}(G_{new})Q(G_{old}|G_{new})}{P(D|G_{old})P_{prior}(G_{old})Q(G_{new}|G_{old})},1\right\}$ **⟨Eq 4⟩** where we use BIC approximation $-\mathrm{BIC}(D,G)=\max_{\theta}(\ln\{P(D|G,\theta)\})-\frac{\ln(R_D)}{2}\mathrm{Dim}(G)+\ln\{P_{prior}(G)\}$ **⟨Eq 5⟩** for $P(D|G)$. The MCMC simulation method is similar to the greedy algorithm [14] in that it consists of an iterative process in which randomly selected local changes to the developing network structure are suggested and then accepted or rejected according to the degree to which they change the quality of the model fit to the data. The Monte Carlo technique accepts the change randomly according to the acceptance probability (Eq 4). A good choice of Q can greatly speed up MCMC convergence on the range of optimal structures. We incorporated this search-space constraint technique [1, 15] into our structure-learning algorithms through an algorithm that creates a "data-derived Proposal Matrix (**DPM**)" defined below. The DPM guides structure learning by defining the sampling proposal distribution $Q(G_{old}|G_{new})$. The structure space can be dramatically shrunk by setting those elements in the DPM which fall below a critical threshold λ equal to zero, thus truncating potential arcs in G considered too unlikely by the statistical measures on which the DPM is based and helping avoid false positives. This amounts to subjecting the search-space to a computationally cheaper "pre-screening," similar to the "sparse candidate" algorithm implemented by Friedman *et al.* [15] to remove implausible structures before engaging more expensive model-based structure-learning techniques. The MCMC method produces an ensemble of models which is used in a structure averaging scheme [16, 17] which should produce a final structure having higher confidence. Without going into detail, the DPM increases the rate of convergence and increases the scalability of structure learning. We have created models with well over 2,500 nodes.

References

1. Friedman, N., et al., *Using Bayesian networks to analyze expression data.* J. Comput. Biol, 2000. **7**: p. 601–620.
2. Hartemink, A.J., et al., *Using graphical models and genomic expression data to statistically validate models of genetic regulatory networks.* Pac Symp Biocomput, 2001: p. 422-433.
3. Imoto, S., et al., *Combining microarrays and biological knowledge for estimating gene networks via Bayesian networks.* J Bioinform Comput Biol., 2004: p. 77-98.
4. Werhli, A. and D. Husmeier, *Reconstructing Gene Regulatory Networks with Bayesian Networks by Combining Expression Data with Multiple Sources of Prior Knowledge.* Statistical Applications in Genetics and Molecular Biology, 2007. **6**(1 Article 15).
5. Thingholm, T.E., et al., *SIMAC (sequential elution from IMAC), a phosphoproteomics strategy for the rapid separation of monophosphorylated from multiply phosphorylated peptides.* Mol Cell Proteomics, 2008. **7**(4): p. 661-71.
6. Shpaer, E., et al., *Sensitivity and Selectivity in Protein Similarity Searches: A Comparison of Smith-Waterman in Hardware to BLAST and FASTA.* Genomics, 1996. **38**: p. 179-91.
7. Consultants, W.G.o. *Health Aspects of Chemical and Biological Weapons*. in *World Health Organization*. 1970. Geneva.
8. Hayes, S.C., and World, M. J, *Adverse reactions to anthrax immunization in a military field hospital.* J. R. Army Med. Corps, 2000. **146**: p. 191-195.
9. Nuhse, T.S. and S.C. Peck, *Peptide-based phosphoproteomics with immobilized metal ion chromatography.* Methods Mol Biol, 2006. **323**: p. 431-6.
10. Schlicker, A., et al., *A new measure for functional similarity of gene products based on Gene Ontology.* BMC Bioinformatics, 2006. **7**(302).
11. Jansen, R., et al., *A Bayesian Networks Approach for Predicting Protein-Protein Interactions from Genomic Data.* Science, 2003. **302**: p. 449-53.
12. Lin, N., et al., *Information assessment on predicting protein-protein interactions.* BMC Bioinformatics, 2004. **5**(154).
13. Hastings, W.K., *Monte Carlo Sampling Methods Using Markov Chains and their Applications.* Biometrika 1970. **57**(1): p. 97-109.
14. Cormen, T., et al., *Introduction to Algorithms*. 2nd ed. 2001, Cambridge, MA: MIT Press.
15. Friedman, N., I. Nachman, and D. Peer, *Learning Bayesian Network Structure from Massive Datasets: The "Sparse Candidate" Algorithm.* Proc. Fifteenth Conference on Uncertainty in Artificial Intelligence (UAI '99), 1999: p. 196–205.

16. Hoeting, J., A.E. Raftery, and D. Madigan, *A Method for Simultaneous Variable and Transformation Selection in Linear Regression.* Journal of Computational and Graphical Statistics 2002. **11**: p. 485-507.
17. Fernandez, C., E. Ley, and M. Steel, *Benchmark Priors for Bayesian Model Averaging.* Journal of Econometrics, 2001. **100**: p. 381-427.
